# Innate Immune Cells' Contribution to Systemic Lupus Erythematosus

**DOI:** 10.3389/fimmu.2019.00772

**Published:** 2019-04-15

**Authors:** Andrés A. Herrada, Noelia Escobedo, Mirentxu Iruretagoyena, Rodrigo A. Valenzuela, Paula I. Burgos, Loreto Cuitino, Carolina Llanos

**Affiliations:** ^1^Lymphatic and Inflammation Research Laboratory, Facultad de Ciencias de la Salud, Instituto de Ciencias Biomédicas, Universidad Autónoma de Chile, Talca, Chile; ^2^Departamento de Inmunología Clínica y Reumatología, Escuela de Medicina, Pontificia Universidad Católica de Chile, Santiago, Chile; ^3^Laboratorio de Enfermedades Autoinmunes Oculares y Sistémicas, Departamento de Oftalmología, Facultad de Medicina, Universidad de Chile, Santiago, Chile; ^4^Departamento de Ciencias Químicas y Biológicas, Facultad de Salud, Universidad Bernardo O'Higgins, Santiago, Chile; ^5^Servicio de Oftalmología, Hospital Clínico Universidad de Chile, Santiago, Chile

**Keywords:** lupus (SLE), innate immunity, dendritic cells, macrophage-cell, innate lymphoid cell

## Abstract

Systemic lupus erythematosus (SLE) is a chronic autoimmune disease characterized by the presence of autoantibodies against nuclear antigens, immune complex deposition, and tissue damage in the kidneys, skin, heart and lung. Because of the pathogenic role of antinuclear antibodies and autoreactive T cells in SLE, extensive efforts have been made to demonstrate how B cells act as antibody-producing or as antigen-presenting cells that can prime autoreactive T cell activation. With the discovery of new innate immune cells and inflammatory mediators, innate immunity is emerging as a key player in disease pathologies. Recent work over the last decade has highlighted the importance of innate immune cells and molecules in promoting and potentiating SLE. In this review, we discuss recent evidence of the involvement of different innate immune cells and pathways in the pathogenesis of SLE. We also discuss new therapeutics targets directed against innate immune components as potential novel therapies in SLE.

## Introduction

Systemic lupus erythematosus (SLE) is a systemic autoimmune disease that affects 20–50 of every 100,000 individuals and whose etiology remains elusive. Whereas early symptoms most frequently involve the skin and joints, disease morbidity and mortality are usually associated with cardiovascular events driven by chronic inflammation, and damage to major organs, particularly the kidneys, the nervous system, hematopoietic organs, and infections derived from immunosuppressant treatments. SLE is also characterized by a myriad of immune system aberrations, including pathogenic autoantibody production and immune complex deposition, and immune system infiltration and inflammation within damaged organs. The main autoantibodies present in the serum of SLE patients are directed against nuclear components [double-stranded DNA (dsDNA), ribonucleoproteins, histones and others]. Systemic tissue damage may arise as a consequence of inflammation caused by direct autoantibody-mediated tissue damage and the deposition of complement-fixing immune complexes (1–3).

Various immune cells and inflammatory mediators have been shown to be harmful players in SLE, especially dysfunctional T and B cells. Hormonal, environmental, and genetic factors are linked to the loss of B- or T-cell tolerance to self-antigens, triggering the activation of both the innate and the adaptive immune system (4, 5). To date, there are no curative treatments for SLE, but current pharmacological approaches for management of SLE have included corticosteroids and immunosuppressive drugs. These drugs can control disease activity, although they have serious, potentially fatal, side effects. B-cell-targeted therapies, including B-cell depletion and blockage of B-cell survival factors, such as B-lymphocyte stimulator (BLyS), have also been developed. While safer than other therapies, B cell-targeted therapies efficacy is still controversial (6). Thus, some studies have shown improvement in lupus while other studies have failed to show any clinical improvement (7, 8). Trial design methodologies including patient's selection, the use of steroids, or short follow up time, can in part explain these discrepancies (9). The results from several trials that are currently underway might clarify these issues (9). Other immune system modulating strategies, including blocking monoclonal antibodies and fusion proteins targeting type 1 IFNs or pro-inflammatory cytokines, such as IL-12 and IL-23, are currently in development (1). Table 1 summarizes the current treatments for SLE.

**Table 1:** Current available treatments in systemic lupus erythematosus.

**Therapy**	**Category**	**Description**	**Condition**	**Type study**	**References**
Glucocorticoids	Steroid hormone	GCR suppress inflammatory mediators and immune cell activity	Lupus nephritis	Clinical trial	(10)
			SLE/lupus nephritis	Systematic review	(11)
			SLE/membranous lupus nephritis	Systematic review	(12)
			SLE/proliferative lupus nephritis	Systematic review	(13)
Methotrexate	DMARDs	Folate analog, inhibits autoimmune T lymphocyte proliferation	SLE	Systematic review	(14)
Hydroxychloroquine	DMARDs	Suppressing activation of Toll-like receptors	SLE	Systematic review	(14)
Azathioprine	DMARDs	Purine analog, inhibits DNA, RNA, and protein synthesis	Lupus nephritis	Systematic review	(11)
			Membranous lupus nephritis	Systematic review	(12)
			Proliferative lupus nephritis	Systematic review	(13)
Mycophenolate mofetil	DMARDs	Inhibits IMPDH, inhibits autoimmune T lymphocyte proliferation	Lupus nephritis	Clinical trial	(15)
			Lupus nephritis	Clinical trial	(16)
			Lupus nephritis	Systematic review	(11)
			SLE	Systematic review	(14)
			Membranous lupus nephritis	Systematic review	(12)
			Proliferative lupus nephritis	Systematic review	(13)
Cyclosporine A	DMARDs	Calcineurin Inhibitors, inhibits autoimmune T lymphocyte proliferation	Lupus nephritis	Clinical trial	(15)
			Lupus nephritis	Systematic review	(11)
			SLE	Systematic review	(14)
			Membranous lupus nephritis	Systematic review	(12)
Tacrolimus	DMARDs	Calcineurin Inhibitors, inhibits autoimmune T lymphocyte proliferation	Lupus nephritis	Systematic review	(11)
			SLE	Systematic review	(14)
			Membranous lupus nephritis	Systematic review	(12)
			Proliferative lupus nephritis	Systematic review	(13)
Cyclophosphamide	DMARDs	Alkylating agent, inhibits autoimmune T lymphocyte proliferation	Lupus nephritis	Clinical trial	(17)
			Lupus nephritis	Clinical trial	(10)
			Lupus nephritis	Systematic review	(11)
			Lupus nephritis	Systematic review	(18)
			Membranous lupus nephritis	Systematic review	(12)
			Proliferative lupus nephritis	Systematic review	(13)
Rituximab^†^	Biologic Response modifiers	Anti-CD20 antibody of B cells	SLE	Clinical trial	(19)
			SLE	Clinical trial	(20)
			SLE	Systematic review	(14)
Belimumab^‡^	Biologic Response modifiers	Anti B-lymphocyte stimulator antibody	SLE	Clinical trial	(21)
			SLE	Clinical trial	(22)
			SLE	Clinical trial	(23)

*DMARDs, Non-biologic disease-modifying antirheumatic drugs; SLE, Systemic Lupus eryhematosus; GCR, glucocorticoid receptor; IMPDH, inosin monophosphate dehydrogenase; IVIg, Intrevenous immunoglobulin*.

† *Off-label used and recommended by clinical guidelines for in SLE and Lupus nephritis*.

‡ *Approved by FDA*.

The innate immune system consists of immune cells, including macrophages, neutrophils, dendritic cells (DCs), basophils, and innate lymphoid cells (ILCs), that circulate in blood or reside in tissues and are poised to respond to pathogens or inflammatory stimuli. Some innate immune cells, such as DCs, can migrate to lymphoid tissues to invoke T and B cell responses and also interface with the other cells in the skin and mucosal epithelia that can produce different cytokines and antimicrobial peptides to influence tissue homeostasis and repair. Recently, the innate immune system has been implicated as a key player in the pathogenesis of SLE. In this review, we will focus on cellular and molecular components of the innate immunity in SLE pathogenesis. We will summarize the current pre-clinical and clinical studies that aim to target innate immunity in SLE.

## Innate Immunity in SLE

### Macrophages

Due to the presence of self-reactive IgG antibodies, SLE development has been commonly associated with dysfunctional adaptive immune responses, especially B-cell responses. This paradigm, however, is shifting, due to rapid advances showing an important role of innate immunity in SLE pathogenesis (24). Studies in patients with SLE and animal models show multiple aberrations in the activation status and secretory functions of circulating and tissue-infiltrating macrophages (25). Such aberrations may be associated with deregulation of T-cell function and autoantibody production in SLE (24, 25). Specifically, a role for macrophages in the pathogenesis of SLE was first proposed following the discovery that SLE macrophages were defective in their ability to clear apoptotic cell debris, thus prolonging exposure of potential auto-antigens to the adaptive immune cells (26, 27). Moreover, activated macrophages are classically categorized in two main groups: classically-activated macrophages (M1), induced by the presence of IFNγ and LPS, that are involved in inflammation and tissue destruction; or alternatively-activated macrophages (M2), induced by IL-4 or IL-13, cytokines that are involved in tissue repair (28). Gene expression profiles from myeloid cells derived from SLE patients and healthy controls have revealed differences in genes that play an important role in macrophage activation and polarization: STAT1 and SOCS3 for M1 are increased; and STAT3, STAT6, and CD163 for M2 are decreased (29). Further, monocyte-derived macrophages from SLE patients show reduced CD163 (M2 marker) and increased CD86 (M1 marker) expression, compared to healthy controls, after treatment with apoptotic cells (30). Thus, M1 and M2 profiles are altered in human SLE patients.

Functional studies in mouse models have shown different roles for M1 and M2 macrophages in SLE. Macrophage depletion in a pharmacological induced-lupus mouse model increases SLE severity (31). Interestingly, adoptive transfer of M2 macrophages reduces SLE severity, but transfer of M1 macrophages increases SLE activity. Thus, M1 macrophages promote tissue damage, while M2 macrophages participate in tissue healing in SLE (31). It seems that skewed M1/M2 responses are also involved in kidney damage during SLE. Nearly 60% of SLE patients develop kidney involvement at some point of the disease, known as lupus nephritis (32). It has been recently demonstrated that, after transient ischemia/reperfusion injury, a non-resolving inflammation develops in mice that are susceptible to developing SLE-like disease. This inflammation is characterized by an increase of M1 vs. M2 macrophages that infiltrate the kidney (33). In summary, defective phagocytosis of apoptotic cells and/or abnormal M1 vs. M2 macrophage polarization can mediate adaptive immune activation and promote autoimmune damage in SLE, suggesting that drugs capable of modulate macrophage function could be a good alternative to develop a strategy against SLE (30).

### Neutrophils

Neutrophils, the most abundant leukocytes in human blood, have been recently linked with SLE. Neutrophils in SLE have abnormal function, including reduced phagocytosis capabilities (34), reduced ability to be cleared by the C1q/calreticulin/CD91-mediated apoptotic pathway (35), and increased oxidative activity (36). Another process that is affected in neutrophils from SLE patients or Lupus-mice models is NETosis (37). First described in 2004, NETosis is a specific form of cell death, characterized by the release of decondensed chromatin coated with antimicrobial, granular proteins into the extracellular space. These neutrophil-derived extracellular traps (NETs) trap and inactivate pathogens (38), but could but also be a source of immunogenic DNA, histones, and neutrophil proteins. In SLE, NETosis is accelerated by the presence of anti-ribonucleoprotein complexes and circulating apoptotic microparticles, which, in turn, activate other immune cell types such as plasmacytoid DCs (pDCs) (39–41). In fact, low-density granulocytes, a specific subset of neutrophils found in SLE patients, show increased NET formation, and these neutrophils are able to infiltrate the kidneys and skin (42). Moreover, patients with active SLE lesions have impaired degradation of NETs due to the presence of DNase I inhibitors and anti-NET antibodies (43). Mechanistically, NETs enriched in oxidized mitochondrial DNA can stimulate production of type I IFNs by direct interaction with the DNA sensor STING (44). In this context, further analysis must be done to establish NETosis as a new potential biomarker or tissue damage predictor in SLE (45).

### Dendritic Cells

In the last decade, our group and others have identified DCs as essential players in the mechanisms underlying SLE, making them attractive therapeutic targets for the fine-tuning of the immune system. Mature DCs can activate T cells. In contrast, tolerogenic (immature) DCs or monocytes, cells able to differentiate to DCs, can promote T-cell hyporesponsiveness, and induce immune tolerance (46). Therefore, tolerogenic DCs or monocytes have emerged as an attractive therapeutic target, because they can induce antigen-specific tolerance without provoking general, widespread immunosuppression. This targeting strategy may reduce or eliminate the development of increased susceptibility to pathogens and opportunistic infections, a commonly-observed side effect of the traditional immunosuppressant drugs currently used in SLE therapy (47–49).

One of the key enzymes that controls monocyte and DC function is heme oxygenase-1 (HO-1), which catalyzes the degradation of the heme group into biliverdin, carbon monoxide (CO), and free iron (Fe^2+^). These byproducts have immunosuppressive and anti-inflammatory activities. In normal conditions, HO-1 is highly expressed in monocytes and DCs, and the products of HO-1 catalysis can contribute to improve tolerance during organ transplantation. HO-1 expression is reduced in monocytes but not in DCs or CD4^+^ T cells from SLE patients or in healthy controls (50). Therefore, HO-1 deregulation may be involved in the initial steps of SLE pathogenesis, rather than in disease progression. HO-1 modulation, as well as CO administration, has emerged as a potential therapy for SLE (51). Genetic or pharmacological modulation of HO-1 and delivery of CO ameliorates disease progression in experimental autoimmune models, such as experimental autoimmune encephalomyelitis, type-1 diabetes, and SLE (52). HO-1 and/or CO can modulate DC and monocyte function (53). CO exposure also decreases B220^+^, CD4^+^ and CD8^+^ T cells in the kidneys and lungs, as well as serum levels of antinuclear antibodies (ANA) of lupus nephritis patients (54). It is also important to mention, as an alternative, non-invasive strategy, a nutritional therapy with extra virgin olive oil increases HO-1 and Nrf-2 protein expression in animal models of SLE and diminishes activation of JAK/STAT, MAPK, and NF-kB pathways that can drive proinflammatory cytokine and chemokine production from many immune cell types (55).

pDCs, first described as the main source of IFNs after viral infection (56), have been linked to SLE development. Early studies showed increased serum levels of IFNα in SLE patients (57, 58). Moreover, genome-wide analysis has identified the IFNα pathway among susceptibility alleles (59, 60). The IFNα inducer, consisting of anti-dsDNA antibodies, and DNA in complex, was identified in the late 1990s (61). pDCs were subsequently identified as the essential origin of IFNα production in SLE (62). IFNα release by pDCs occurred mainly at early-stages during disease, since pDCs from late-stages disease are unable to produce IFNα in the MRL/Mp-Faslpr (lpr) lupus mouse model (63). pDCs numbers are diminished in blood of SLE patients, but pDCs accumulate in the inflamed or damaged skin of lupus patients, suggesting that the reduced numbers of pDCs in blood could be in part explained by the rapid migration to inflamed tissues (64, 65). By using different lupus mouse models, such as (NZBxNZW)F1 mice, BXSB.DTR mice or Tlr7.Tg animals, it has been seen that depletion of pDCs ameliorates SLE manifestations and inflammation, suggesting that targeting the function or accumulation of pDCs in tissues could be a viable therapy to ameliorate SLE (66–68).

### Basophils

Basophils are the rarest immune cell population in the blood, representing only 1% of circulating leukocytes. Initially implicated in allergic events and parasite infections, increasing evidence has suggested a role of basophils in SLE (69, 70). Basophils are recruited into skin lesions of SLE patients, where they are implicated in promoting tissue damage (71). Data from a retrospective clinical study suggest that blood basophils could be potentially used as a biomarkers of disease activity in SLE (72, 73). By using the Lyn^−/−^ mice, that develop a lupus-like disease late in life, Charles et al. showed that basophils are key players in promoting inflammation and supporting ANA production by B cells (69). Although initially controversial (74), this concept of basophils and SLE has been expanded in the recent years by studies showing that basophils derived from human SLE patients are able to promote antibody production by B cells and support IL-17-producing T_H_17 differentiation of T cells *in vitro* (70). In line with the importance of basophils in SLE development, a very recent study has shown that Prostaglandin D_2_ (PGD_2_), an important inflammatory mediator, is elevated in plasma from SLE patients and through the interaction with PGD_2_ receptors expressed by blood basophils, leads to basophils accumulation in secondary lymphoid organs (75). Moreover, PGD_2_ receptors blockade leads to the reduction of basophils migration into secondary lymphoid organs, dampening lupus-like disease activity in Lyn^−/−^ mice (75). Whether basophils could be a suitable therapeutic target in SLE remains to be evaluated.

### Innate Lymphoid Cells

Described about 10 years ago, ILCs represent an emerging family of innate immune cells. ILCs lack of antigen-specific receptors that are expressed by B and T cells, have a lymphoid-like morphology, and share cytotoxic and immunomodulatory capacities with cytotoxic CD8^+^ and helper CD4^+^ T cells (76). Current classification of ILCs is based on their transcription factors and cytokine production profile: NK cells, expressing the transcription factor EOMES with unique cytotoxic capacities; group 1 ILCs that express the transcription factor T-bet and produce IFN-γ; group 2 ILCs that express RORα and GATA3 and produce type 2 cytokines (e.g., IL-4, IL-5, IL-9, and IL-13); and group 3 ILCs that express the transcription factor RORγt and produce IL-17A and IL-22 (77, 78). Recently, a link between ILCs and SLE has been found in an animal model, where reduced numbers of renal-infiltrating ILC2s were observed in the MRL/Mp-Faslpr (lpr) lupus mouse model (79). The reduction was also observed as disease progresses. More interestingly, restoring ILC2 numbers by treatment with IL-33 reduces immune cell infiltration in the kidney glomerulus and improves survival (79). These findings are consistent with a recent study showing that circulating ILC1s and ILC3s are increased, whereas ILC2s numbers are reduced in SLE patients (80). Although more studies are needed to confirm and expand upon these observations, manipulation of the numbers, and functions of ILCs could be a good candidate for future therapeutic approaches.

### Molecular Regulators of Innate Immunity: The Complement System, Cytokines, and Toll-Like Receptors (TLRs)

Another important innate immune component is the complement cascade. Consisting of more than 30 proteins, the activation of the complement cascade leads to the production of opsonins and chemoattractant cytokines, promotes the production of antibodies, and drives the clearance of immune complexes, apoptotic cells and debris (81). Individuals who are deficient in the early complement proteins C1 and C4 are highly susceptible to developing SLE, with C1q deficiency a stronger genetic predictor to the disease (93% of individuals with C1q deficiency, and 75% of individuals with C4 deficiency present SLE-like symptoms) (82). Mice deficient for C1q or C4 are also predisposed to develop SLE-like disease (83, 84). Two hypotheses have emerged to explain these observations. One called the “waste disposal hypothesis,” suggests that the complement cascade eliminates apoptotic cells and debris, therefore preventing the accumulation of self-antigens that could activate adaptive immune cells (83). The second “tolerance hypothesis” states that the complement cascade is important to generate B-cell tolerance by eliminating autoreactive B cells (85). Interestingly, deficiency of C3, another early component of the cascade, is not associated with SLE development (86). Recently, a study from Botto's group suggested that C1q, but not C3, can promote metabolic changes in CD8^+^ T cells, regulating their function and reducing autoimmunity damage, thereby partially clarifying the discrepancy between C1q and C3 deficiency (87). These observations suggest C1q as a potential therapeutic target, but more studies are needed to evaluate this idea.

There is also growing evidence supporting the pathogenic role of cytokines in this disease. Examples of these cytokines include BLyS, IL-6, IL-17, IL-18, type I IFNs, and TNF- α (88). Cytokines regulate and control the immune system. In SLE, several of these cytokines are overexpressed and contribute to the pathogenesis of disease. Cytokine inhibition has been successfully used to treat other rheumatic and autoimmune diseases, and several cytokines are currently being investigated to determine whether inhibition would be therapeutic in lupus. Several cytokines are undergoing clinical trials, including TNF-α, IL-1, IL-6, IL-10, IL-15, IL-17, IL-18, and IL-23. While current trials have not proven efficacy (Table 1), cytokine targeting is still a promising strategy to ameliorate SLE progression (89).

**Figure 1:**
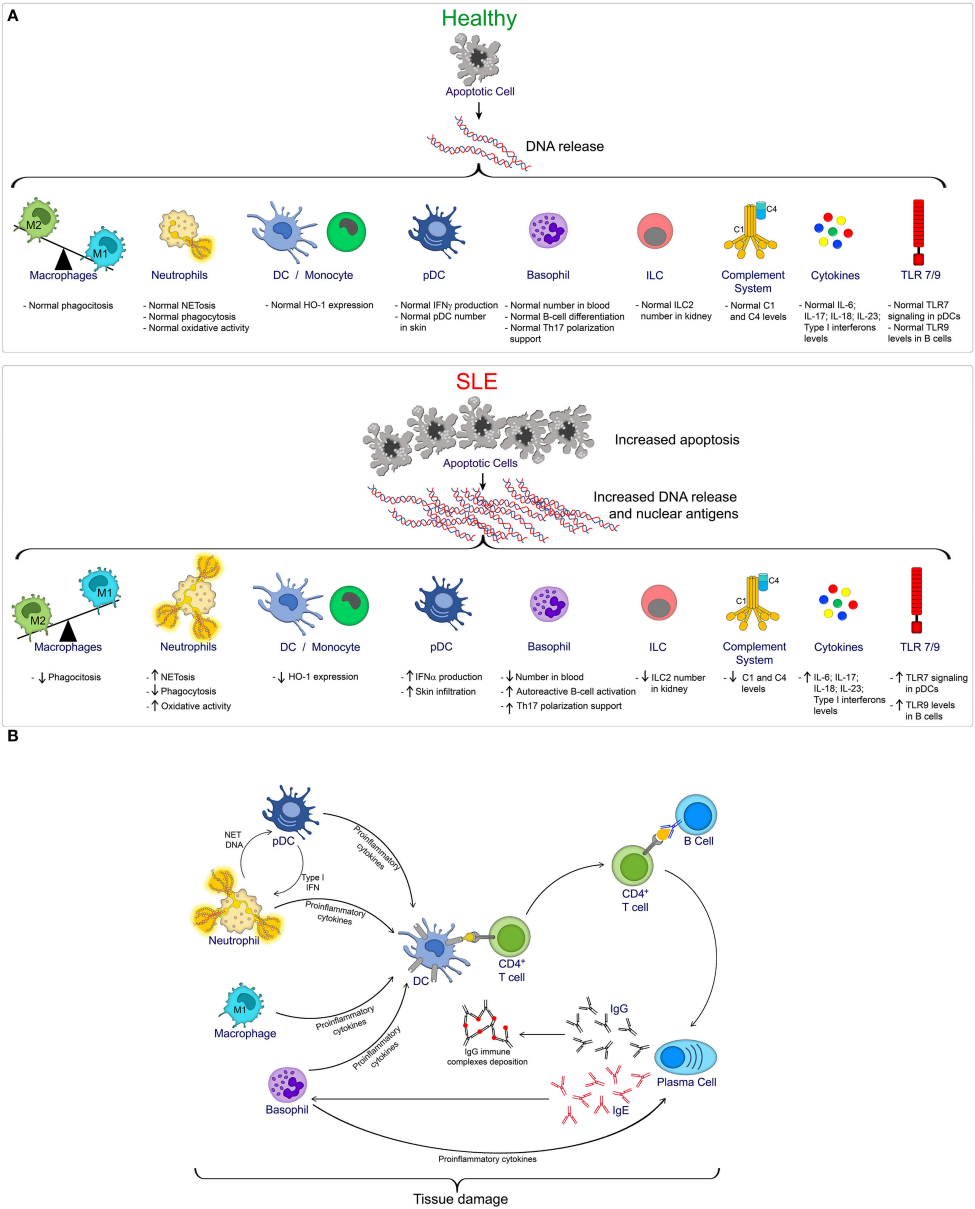
**(A)** Overview of innate immune cells and pathways compromised during SLE progression. In homeostatic condition (Healthy), controlled apoptotic cell death is rapidly cleared, reducing the exposition of nuclear antigens, and reducing the risk of autoimmunity. However, increased apoptosis, as observed during SLE, together with a defective clearance, favors the exposition of DNA and nuclear antigens, promoting the activation of multiple innate immune cells, and pathways that contribute to SLE pathogenesis. **(B)** Brief summary of innate and adaptive immune cell interaction during SLE.

Toll-like receptors (TLRs), a group of glycoproteins that function as surface or endoplasmic trans-membrane receptors, are involved in the innate and adaptive immune responses to exogenous pathogenic microorganisms. TLRs are widely expressed in immune cells (neutrophils, monocytes/macrophages, lymphocytes and DCs), and their activation leads to an inflammatory response by recognizing pathogen and danger-associated molecular patterns (PAMPs and DAMPS). TLRs are a key link between infection, injury and inflammation (90). Although TLR-mediated inflammation is an important aspect of host defense, it is also associated with the pathogenesis of SLE (91). Since self-DNA and self-RNA can form protein complexes and serve as TLR9 and TLR7 ligands, respectively, TLR stimulation may contribute to activation and/or modulation of the immune response (92, 93). The numbers of human peripheral blood B cells and monocytes expressing TLR9 are elevated in patients with SLE, and this increased expression correlated with increased complement function and SLE disease severity (90, 94). Regarding TLR7, a recent study has shown that pDCs derived from SLE patients have increased IFN-α production after TLR7 stimulation compared to pDCs derived from healthy donors (95). Mechanistically, TLR7 is retained in late endosome/lysosome compartments in pDCs from SLE patients, increasing TLR7 signaling and IFN-α production (95). Moreover, in a systematic review and meta-analysis, *TLR7* and *TLR9* polymorphisms were shown to be associated with the development of SLE in Asian populations (96). Additionally, increased expression of other TLRs, such as TLR2, TLR3, TLR4, or TLR5 has been observed in immune cells or biopsies of SLE patients, and studies in animals models have suggested the importance of these TLRs in promoting SLE pathogenesis (97–102). The accumulation of evidence for TLRs in autoimmunity has opened the door for potential therapeutic interventions directed toward the modulation of TLRs and their signaling pathways (103). A summary of innate immune components involved in SLE pathogenesis and the interaction between innate and adaptive immune cells during SLE is shown in Figure 1.

## Concluding Remarks

Despite the importance of adaptive immune responses mediated by B and T cells during SLE pathogenesis, the role of innate immune components has been only recently addressed. Now, we know that a complex network of innate and adaptive immune cells interactions occurs during SLE. This complexity allows scientists and clinical researchers to explore a wide source of possible new therapeutics targets. We predict that new studies will continue to show the importance of innate immune components during SLE. These analyses will provide the groundwork for new therapeutic approaches that modulate innate immune cells accumulation or function as new strategies to limit or ameliorate SLE pathology.

## Author Contributions

AH, LC, and CL wrote the manuscript. NE made the figure. RV made the table. AH, NE, MI, RV, PB, LC, and CL read, discussed, and revised the manuscript. All authors listed have made a substantial, direct and intellectual contribution to the work, and approved it for publication.

### Conflict of Interest Statement

The authors declare that the research was conducted in the absence of any commercial or financial relationships that could be construed as a potential conflict of interest.
